# A novel bacterial consortium isolated from long-term plastic-contaminated soil exhibits efficient biodegradation of polyvinyl chloride microplastics

**DOI:** 10.1186/s12934-026-03011-z

**Published:** 2026-05-05

**Authors:** Nawal Magdy, Mahmoud S. Maher, Ahmed Mohamed Soliman, Tarek A. A. Moussa, Hoda M. Shehata

**Affiliations:** 1 Botany and Microbiology Department, Faculty of Science, Cairo University, Giza, 12613 Egypt; 2 Department of Zoology, Faculty of Science, Cairo University, Giza, Egypt

**Keywords:** Polyvinyl chloride microplastics, Biodegradation, *Stutzerimonas sp.*, *Glutamicibacter nicotinae*, Bacterial consortium, 16s rRNA

## Abstract

**Background:**

Polyvinyl chloride (PVC) is one of the most widely used synthetic polymers globally, and its continuous accumulation in natural ecosystems has emerged as a critical environmental and public health concern. Recently, microbial degradation has been recognized as an efficient and eco-friendly strategy for mitigating plastic pollution. Despite increasing interest, knowledge of bacteria capable of efficiently degrading PVC microplastics (PVC-MPs) remains limited. This gap highlights the urgency of exploring novel bacterial candidates for effective PVC biodegradation.

**Methodology:**

In this study, soil samples collected from plastic-contaminated sites were utilized to isolate PVC-degrading bacteria using enrichment culture techniques. Bacterial isolates showing potential interaction with PVC were selected and molecularly identified. In addition, their efficacy in degrading PVC-MPs was further confirmed through a combination of analytical and spectroscopic techniques, including scanning electron microscopy (SEM), Fourier-transform infrared spectroscopy (FTIR), thermogravimetric analysis (TGA), and gas chromatography–mass spectrometry (GC–MS).

**Results:**

The two isolates identified as *Stutzerimonas sp.* NH2 and *Glutamicibacter nicotinae* NH27 displayed markedly different PVC biodegradation efficiencies. Strain NH2 achieved a PVC-MPs weight loss of 23.41 ± 0.93%, whereas strain NH27 exhibited a lower degradation efficiency of 5.87 ± 2.16%. Notably, the consortium composed of both strains in equal volumes resulted in a greater PVC-MPs weight loss of 26.84 ± 0.94%, representing a significant increase in PVC-MPs degradation compared with each strain alone (*p* < 0.05). SEM analysis revealed pronounced morphological alterations on PVC surfaces following bacterial exposure, including cracks, fissures, and grooves. FTIR spectra demonstrated a substantial reduction in the intensities of some functional groups, which could be attributed to PVC degradation. TGA analysis showed a measurable decline in thermal stability, further suggesting chemical structural modifications due to bacterial activity. Additionally, GC–MS analysis detected potential degradation products, providing clear chemical evidence of bacterial-driven PVC degradation.

**Conclusion:**

This study reports, for the first time, the potential involvement of *Stutzerimonas* sp. NH2 and *Glutamicibacter nicotinae* NH27 in the transformation of PVC microplastics. The findings also provide initial insights into the combined activity of these two strains on PVC-MPs, supported by multiple physicochemical analyses. These results contribute to the growing understanding of microbial interactions with PVC microplastics and highlight the potential of these bacteria for future bioremediation studies.

**Graphical Abstract:**

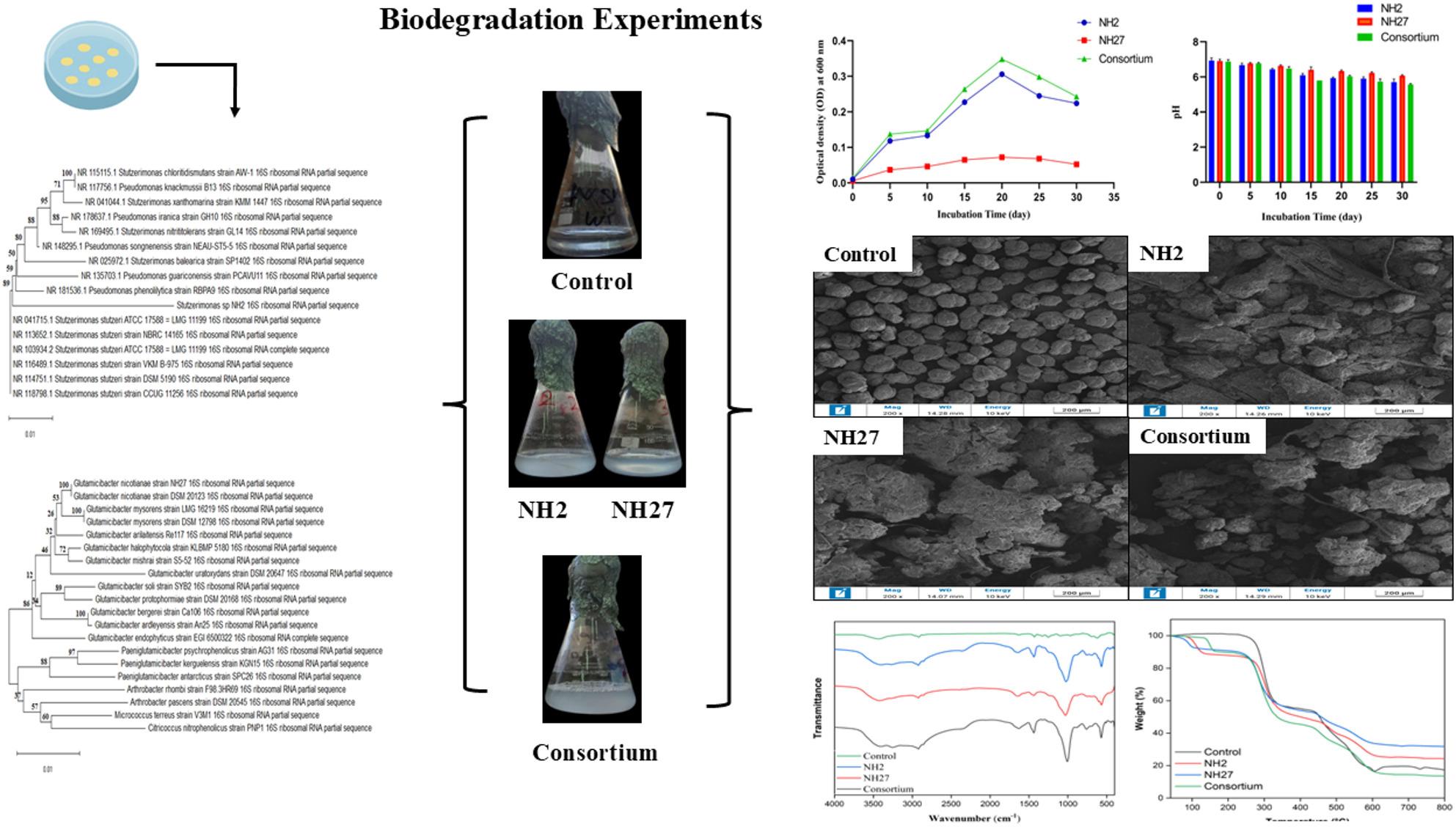

## Background

Plastic is a crucial material widely used across many sectors, and its production is growing in tandem with the rapid expansion of the global economy [1]. It is estimated that worldwide plastic manufacturing reaches 400 million tons annually, with average annual growth rates exceeding 4% [2]. Most discarded plastics are not recycled and are frequently transformed into microplastics (MPs), which measure less than 5 mm. These particles are widely dispersed in the environment, including marine, freshwater, and soil systems [3]. MPs are pervasive in the aquatic environment, infiltrating the water column and being ingested by marine organisms, penetrating the skin, and entering the circulatory system [4]. This uptake can cause harmful impacts in marine animals, including oxidative stress, immunotoxicity, and developmental and reproductive toxicity [5, 6]. Therefore, the threat of MPs contamination impacts the entire ecosystem and poses a considerable risk to human health [7]. Consumption of fish containing MPs is a major pathway for human exposure, particularly in communities that depend predominantly on aquatic animals as a primary protein source [8].

Focusing on specific polymers, Polyvinyl chloride (PVC) is one of the most prevalent types in chemical manufacturing [9]. Although assessments identify PVC as the most hazardous polymer, it remains the largest global market for plastics [10, 11]. PVC is widely utilized in construction materials, home furnishings, plastic pressure pipe systems, and the water and sewage sectors [12].

Recent studies have revealed that specifically PVC-MPs may induce several harmful effects, including intestinal damage, hepatotoxicity, neurotoxicity, growth inhibition, immunoregulation, and oxidative stress in various species of aquatic animals, such as *Clarias gariepinus*, *Cyprinus carpio*, *Dicentrarchus labrax*, and *Danio rerio* [13–17]. Furthermore, exposure to PVC-MPs constitutes an immense threat to human health [3]. These particles can infiltrate the intestinal wall and enter the lymphatic system and bloodstream, causing an excessive and inappropriate activation of immune cells [18].

Thus, finding an environmentally friendly, sustainable, and economically viable approach to disposing of this plastic waste is crucial [19]. The prevalent methods for MPs degradation include chemical, physical, and biological processes. In contrast to chemical and physical treatments, biodegradation, particularly microbial biodegradation, has the advantages of low cost, environmental sustainability, and ecological friendliness [20, 21]. Several studies have investigated the ability of various bacterial species to degrade plastics and utilize their polymers to survive, such as *Alcanivorax borkumensis* [22], *Bacillus cereus* [23], *Vibrio sp.* [24], *Streptomyces sp.* [25], *Rhodococcus sp.* [26, 27], *Bacillus sp.* [28–30], and *Exiguobacterium sp.* [31]. Due to its ubiquity, PVC disposal warrants heightened priority, and several studies have effectively used bacteria to biodegrade its particles [32–34]. However, the technology for biodegrading MPs remains nascent; only a limited number of plastic-degrading species and enzymes have been identified [35].

In this study, we sought to isolate and characterize novel bacterial strains capable of degrading PVC microplastics and to assess their biodegradation efficiency, both individually and in combination, to identify promising candidates for the sustainable management of this persistent plastic pollution. This study offers comparative insights into the behavior of single vs. co-cultures during the degradation of PVC-MPs, hence enhancing the limited understanding of microbial interactions in the breakdown of extremely refractory polymers like PVC.

## Materials and methods

### Polyvinyl chloride microplastics

PVC-MPs were obtained from Sigma Aldrich (CAS-No.9002-86-2). MPs particles were white powder with an average molecular weight (Mw:48000), particle size ≈ 100 μm, and a density of 1.4 g/mL at 25 °C. PVC-MPs were sterilized by placing them in glass Petri dishes and soaking in 99.9% anhydrous ethanol, followed by overnight drying in an oven at 50 °C [36].

### Sample collection

**Fig. 1:**
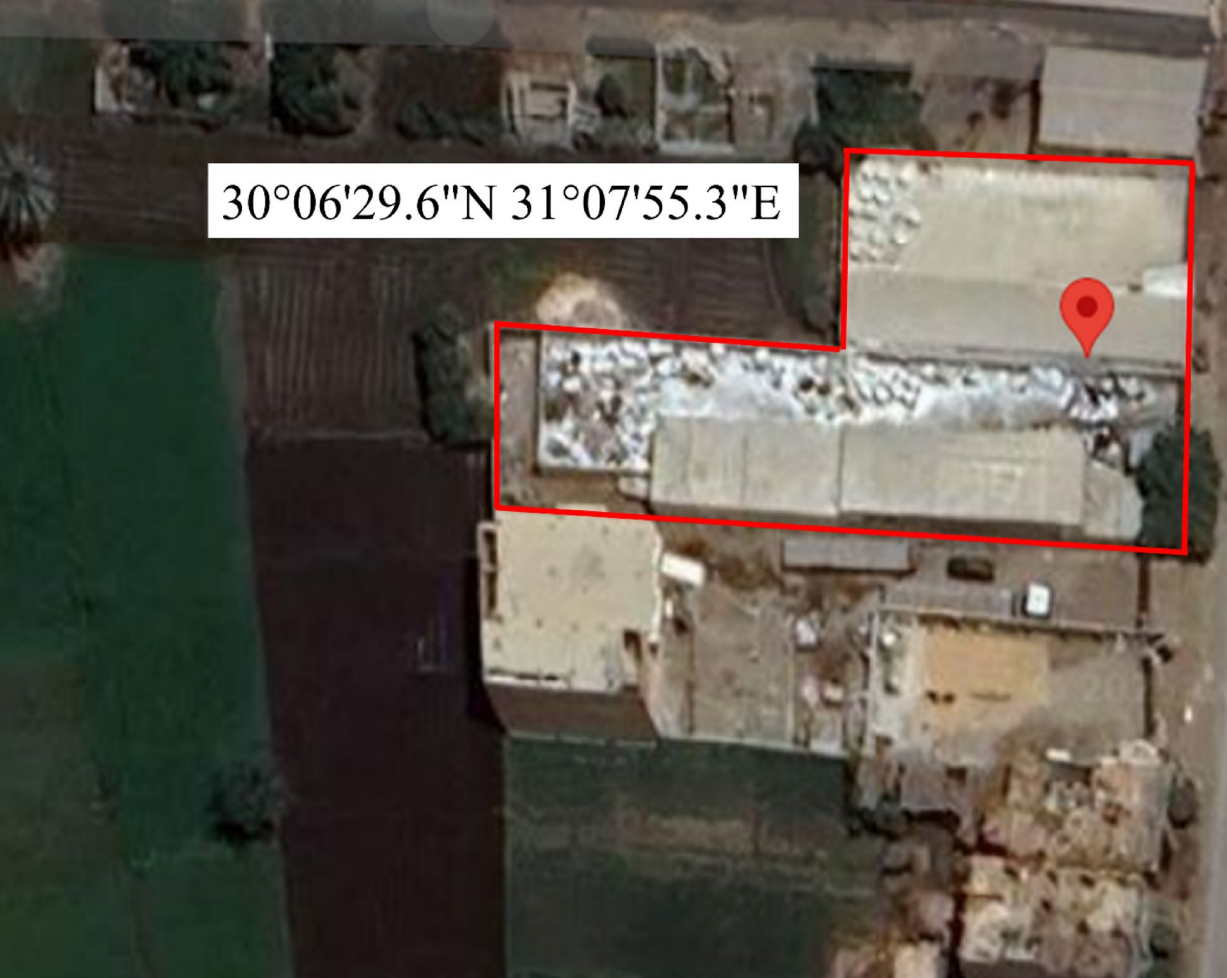
Map showing the location of the factory site used for soil samples collection

Soil samples were collected from two locations: areas surrounding a local plastic manufacturing factory in Giza and places within the factory premises (Fig. 1). The factory-site soils had been exposed to plastic waste for nearly a decade, reflecting typical conditions of plastic contamination and a greater probability of the existence of bacteria capable of adapting to such an environment. All soil samples were stored and handled under sterile conditions prior to use in the isolation of PVC-MPs-degrading bacteria.

### Isolation of PVC-MPs-degrading bacteria

PVC-MPs-degrading bacteria were isolated using the enrichment culture technique. Mineral Salt Medium (MSM) was used to provide essential salts while forcing bacteria to utilize PVC-MPs as the sole carbon source. Soil samples were suspended in sterile normal saline and inoculated into 200 mL of MSM of the following composition per liter: NaH_2_PO_4_, 1.39 g, KH_2_PO_4_, 1.0 g, (NH4)_2_SO_4_, 0.5 g, MgCl₂⋅6 H₂O, 0.1 g, Ca (NO3)_2_⋅4H_2_O, 0.05 g, and 1.0 mL of trace element solution. Sterilized PVC-MPs (0.1 g/L) were added as the sole carbon source. Enrichment cultures were incubated at 37 °C with shaking at 120 rpm for 4 weeks in total, with cultures transferred to fresh MSM containing PVC-MPs every 14 days to enrich for bacteria capable of utilizing PVC as the sole carbon source. After the two enrichment cycles, samples were serially diluted, and 50 µL aliquots of the 10⁻² and 10⁻³ dilutions were plated onto MSM agar containing PVC-MPs as the sole carbon source. Plates were incubated at 30 °C for 3–5 days. Morphologically distinct bacterial colonies were subsequently purified by repeated subculture and maintained on slanted nutrient agar for further characterization and biodegradation studies [23, 37, 38].

### Molecular identification of the most promising isolates

The total genomic DNA was extracted using the QIAamp DNA Mini Kit (Qiagen, Germany GmbH) with modifications to the manufacturer’s recommendations. The DNA samples were amplified using primer sets for 16S rRNA 27 F (5’- AGAGTTTGATCMTGGCTCAG − 3’) and 1492 R (5’ TACGGYTACCTTGTTACGACTT − 3’). The final volume of the PCR system was 25 µl each, containing 12.5 µl of EmeraldAmp Max PCR Master Mix (Takara, Japan), 1.0 µl of each primer, 5.5 µl of water, and 5 µl of DNA template. The reaction was performed in an Applied Biosystem 2720 thermal cycler. The PCR conditions were as follows: 1 cycle of initial denaturation (94 °C for 5 min), 30 cycles of annealing and extension (94 °C for 30 s, 56 °C for 1 min, 72 °C for 1.2 min), and final amplification of DNA, Extend 1 cycle (72 °C for 12 min**)** [39]. PCR products were purified using QIAquick PCR Product Extraction Kit (Qiagen, Valencia) and sequenced using the Sanger sequencing platform. The reads were trimmed and assembled, and contigs for each isolate were generated. Then, they were compared against reference 16 S rRNA gene sequences available in GenBank using the nucleotide BLAST tool. The phylogenetic tree was constructed using the neighbor-joining method in MEGA X software [40] with 1000 bootstrap replicates.

### Biodegradation experiments setup

Biodegradation experiments were conducted to evaluate the potential of the isolated bacterial strains to degrade PVC-MPs, both individually and as a consortium. Sterilized PVC-MPs (1.0 g/L) were added to flasks containing 100 mL of Mineral Salt Medium (MSM) as the sole carbon source. Each flask was inoculated with either a single bacterial strain or an equal-volume mixture of both strains to form the consortium (final inoculum volume equals 1 ml in all cases). Flasks without bacterial inoculation were included as a control. All flasks were then incubated at 37 °C while shaking at 150 RPM for 30 days. All assays were performed in triplicate [41].

### Analysis procedures

#### Microbial growth and pH determination of the medium

The optical density (O.D. at 600 nm) and pH were monitored every 5 days for 30 days. The data of OD_600_ reflects the bacterial growth in the medium, while the measurements of pH reflect the bacterial metabolism in the designed medium [42]. The OD of the inoculated flasks at time 0 was measured and is reported.

### Determination of dry weight, reduction rate, and half-life of residual PVC-MPs

When cultivation was terminated after 30 days, the MPs samples were collected by filtration using Whatman No. 1 filter paper (pore size ~ 11 μm), and the bacterial biofilm was removed from their surface by washing for 4 h with a 2% (w/v) SDS solution with gentle manual shaking. The samples were then dried overnight at 50 °C in an oven. The degradation degree was determined by using a four-decimal balance (0.0001 g) to determine the weight change of the dried MPs [43]. The following equation was used to estimate the percentage weight loss of PVC-MPs [44].

$$ \:{\mathrm{Weight}}\:{\mathrm{loss}}\: = \frac{{W0 - \:W}}{{W0}} \times \:100 $$

Where W0 and W are the initial weight (g) of PVC-MPs and the residual weight (g) of PVC-MPs, respectively.

To determine the removal rate constants of MPs polymers, the data were further processed using a first-order kinetic model as follows [45]:

$$\:\mathrm{K}\:=-\frac{1}{t}\:\left(\mathrm{l}\mathrm{n}\:\frac{w}{w0}\:\right)$$

Where K is the daily removal rate constant of polymer absorption, t is the time in days, W0 is the initial weight of PVC-MPs (g), and W is the residual weight of PVC-MPs (g). After generating the removal rate constant of the MPs polymer, we calculated the half-life (t_1/2_) as follows [45]:

t1/2 = ln2 \ K.

### Scanning electron microscopy (SEM)

Scanning electron microscopy (FEI-SEM, Inspect S50, Netherlands) was performed at a voltage of 15–30 kV to screen alterations in the surface morphology of PVC-MPs (pits, cracks, and holes) following 30 days of culturing with degrading bacteria. That was achieved using the air-dried samples, which were coated with platinum (LICA EM ACE 600).

### Fourier transform infrared (FTIR) analysis

FTIR spectroscopy (FTIR-4100 type A) was used to examine and identify the alterations in the composition and functional groups of PVC-MPs samples cultivated with bacteria and those without inoculation. FTIR spectra were analyzed at a resolution of 4 cm⁻¹ within the 4000–500 cm⁻¹ range.

### Thermogravimetric analysis of PVC-MPs

Thermogravimetric analysis (TGA/DSC 3+/1600 HT, Mettler-Toledo, Switzerland) was used to evaluate the heat stability of PVC-MPs samples before and after degradation by various bacterial treatments across a temperature range of 50 °C to 800 °C. Approximately 10 mg of dried PVC-MPs were evaluated by thermogravimetric analysis in a nitrogen atmosphere (gas flow: 40 ml/min). Thermograms were measured from 50 °C to 800 °C at a heating rate of 10 °C/min.

### Gas chromatography mass spectroscopy (GC-MS)

To identify potential degradation products generated by the bacterial consortium, GC–MS analysis was performed as described by Bansal et al. [36], with some modifications. Firstly, the bacterial cultures were centrifuged at 13,000 rpm for 10 min to remove residual cells and MPs. The supernatant was then filtered through Whatman No. 2 filter paper. Extraction of the degradation products was performed with diethyl ether; each filtrate was dissolved in 10 mL of the solvent in a separating funnel. The extracted samples were dried under a gentle stream of nitrogen at 40 °C to remove the solvent. For derivatization, each dried sample was resuspended in 50 µL of bis(trimethylsilyl)trifluoroacetamide (BSTFA) containing 1% trimethylchlorosilane (TMCS) and 50 µL of pyridine to convert polar functional groups into trimethylsilyl (TMS) derivatives prior to GC–MS analysis. GC–MS was performed using an Agilent 7890B gas chromatograph coupled with a 5977 A mass spectrometer. Separation was achieved on an HP-5MS column (15 m × 0.25 mm i.d., 0.25 μm film thickness) using hydrogen as the carrier gas at 1.1 mL/min in splitless mode, with an injection volume of 1.0 µL. The oven temperature program was: 40 °C for 1.0 min, ramped at 10 °C/min to 200 °C (hold 1 min), ramped at 20 °C/min to 220 °C (hold 1.0 min), and ramped at 30 °C/min to 300 °C (hold 5 min), with injector and detector temperatures set at 280 °C and 300 °C, respectively. Mass spectra were recorded by electron ionization at 70 eV over m/z 50–800, with a solvent delay of 2.4 min, a source temperature of 230 °C, and a quadrupole temperature of 150 °C. Degradation products were identified by comparing their fragmentation patterns with reference spectra in the Wiley and NIST Mass Spectral Libraries.

### Statistical analysis

All experiments were performed in triplicate, and the results are expressed as mean ± standard deviation (SD). Graphs were generated using GraphPad Prism 8 (GraphPad Software Inc., San Diego, CA, USA). Statistical analyses were carried out using SPSS version 20 (IBM SPSS Statistics for Windows), employing one-way analysis of variance (ANOVA) followed by Duncan’s Multiple Range Test to compare means. Differences were considered statistically significant at a P value < 0.05.

## Results

### Bacterial isolation and identification

**Fig. 2:**
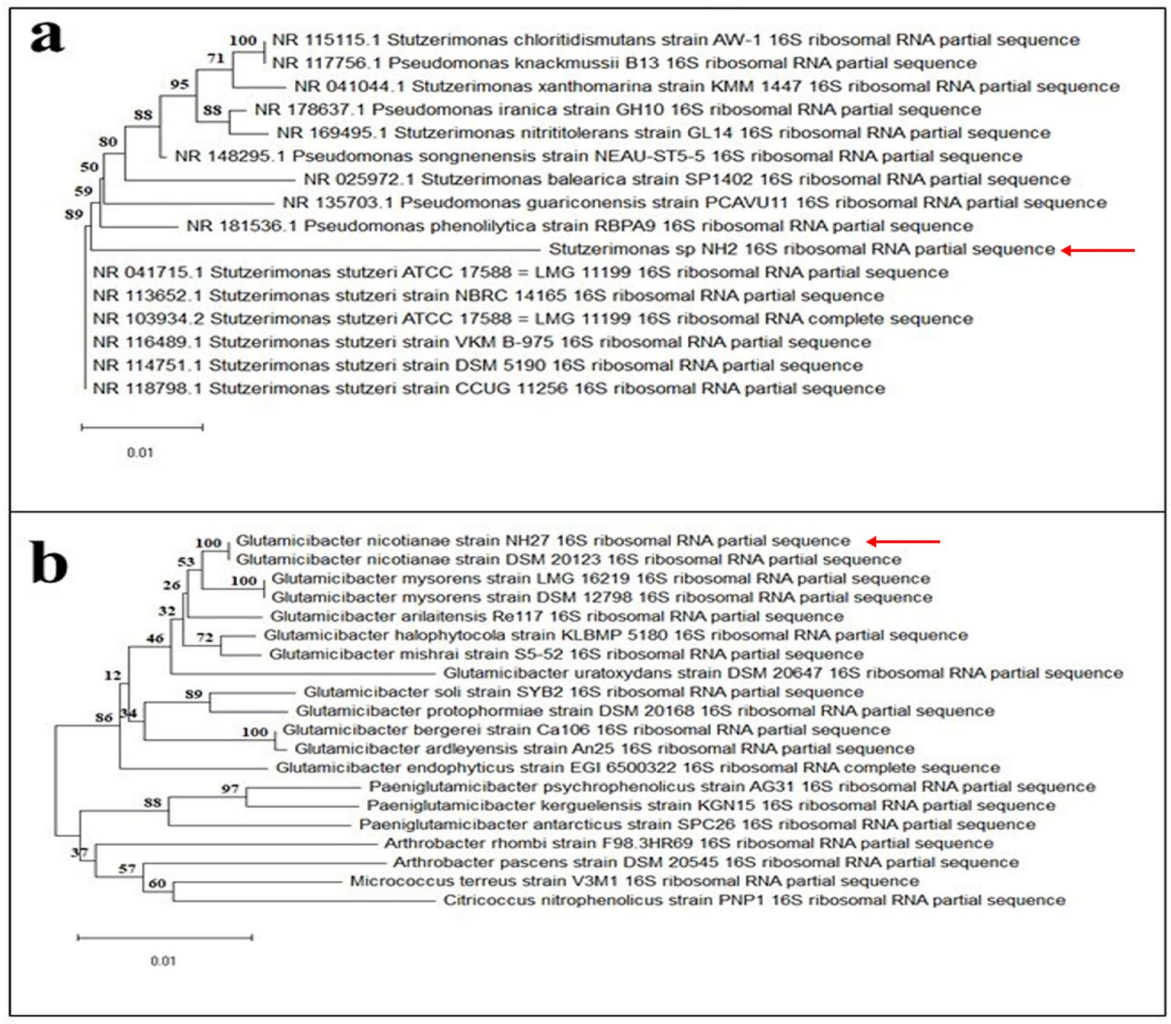
Phylogenetic tree based on 16 S rRNA gene homology sequences constructed using the neighbor-joining method of the MEGA X software, showing (**a**) the relationships between *Stutzerimonas sp* strain NH2 and the most closely related species. (**b**) the relationships between *Glutamicibacter nicotianae* strain NH27 and the most closely related species

A continuous dilution method was employed for 30 days, yielding 30 morphologically distinct bacterial isolates from soil samples potentially containing PVC-MPs. Five colonies with apparent growth differentiation and advantages, such as faster growth and higher abundance compared to other isolates, were selected for their biodegradation potency of PVC-MPs in the screening experiment. During the screening experiment, cell growth was monitored by optical density (OD), an indirect indicator of PVC-MPs biodegradation. Based on screening results, two strains were selected as the most potent bacteria for degrading PVC-MPs and were subjected to molecular identification. The 16 S rRNA gene of each selected strain was amplified and sequenced. The reads were assembled, and the contig sequence was submitted to the NCBI GenBank database under the accession numbers PX642999 for isolate NH2 and PX643024 for isolate NH27. Among the two bacterial cultures, strain NH2 was 95.01% like *Stutzerimonas stutzeri* NR_118798.1, and strain NH27 was 99.64% similar to *Glutamicibacter nicotianae* NR_026190.1. Therefore, we can consider that bacterial culture NH2 is *Stutzerimonas sp*, while bacterial culture NH27 is *Glutamicibacter nicotianae*. The phylogenetic tree was constructed to illustrate the relationships between these isolates and their closest related species, as shown in Fig. 2.

### Bacterial growth and ph changes across the incubation period

**Fig. 3:**
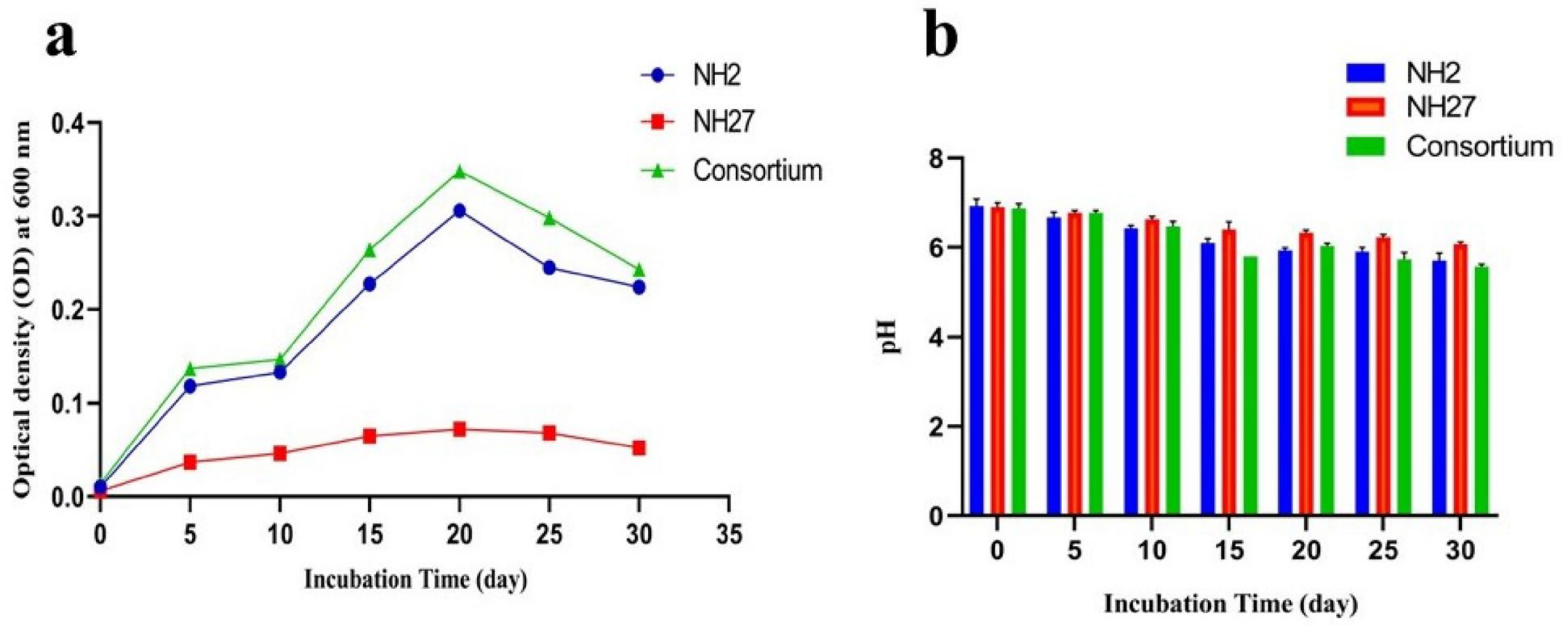
Growth curves of the two bacterial strains (*Stutzerimonas* sp. NH2 and *Glutamicibacter nicotinae* NH27), and the consortium during the 30-day incubation period (**a**). Changes in the medium pH during biodegradation of PVC-MPs (**b**)

The growth rates of each bacterium alone and an equal combination of two strains were measured every 5 days. Four treatments (control, strain NH2, strain NH27, and consortium) were conducted over 30 days. From the first day to the fifth day, exponential growth was observed in both the single bacteria and the bacterial complex. The Optical density (OD) value at 600 nm of the single bacteria NH2 and NH27 increased from 0.01 to 0.11 and 0.03, respectively. In contrast, the OD_600_ value of the combined bacteria grew to 0.14. From 5 days to 15 days, single bacteria NH2 and NH27 increased up to 0.22 and 0.06, while bacterial consortia grew to 0.26. The growth increased until the maximum was reached at the 20th day, with NH2 and NH27 at 0.30 and 0.07, respectively, while the consortium was 0.35. After exposure to PVC-MPs for 20 to 30 days, the growth of NH2, NH27, and their consortium decreased to 0.22, 0.05, and 0.24, respectively, as shown in Fig. 3a. The pH changes of the culture medium in the control and treatment groups during the 30-day PVC-MPs biodegradation process are illustrated in Fig. 3b. The initial pH of the culture medium was approximately 6.9 for all treatments. The biodegradation of PVC–MPs markedly decreased the pH of the medium in NH2 and NH27, and in the consortium, making it more acidic. Continuous decrease in pH from day 0 to day 30, where on the 30th day, the pH value in the culture medium of NH2 and NH27 was 5.7 and 6.06, respectively, while the consortium had the lowest pH value of 5.56.

### Dry weight loss, degradation rate, and half-life of PVC-MPs

The biodegradation efficiency of the bacterial isolates and their consortium was further assessed by comparing the dry weight loss of PVC-MPs. After 30 days of single-strain treatment, the weight-loss percentages for PVC-MPs treated with strains NH2 and NH27 were 23.41 ± 0.93% and 5.87 ± 2.16%, respectively. The consortium of two strains (in equal volumes) has a weight-loss percentage of 26.84 ± 0.94%.

The removal rate constants for single treatments were 0.00889 ± 0.0004 day^− 1^ and 0.00202 ± 0.0008 day^− 1^ for NH2 and NH27, respectively, while that for their consortium was 0.01042 ± 0.0004 day^− 1^. The half-lives were 78.07 ± 3.58, 389.94 ± 187.49, and 66.6 ± 2.77 days, respectively, for NH2, NH27, and combination (Table 1).

**Table 1:** Effect of different bacterial treatments on weight loss percentage, removal rate, and estimated half-life of PVC-MPs after 30 days of biodegradation

Treatment	Weight (g)			Removal constant (K) day⁻¹	Half-life (days)
	Initial	Final	Loss (%)		
*Stutzerimonas* sp. (NH2)	0.100	0.0766 ± 0.0009^b^	23.41 ± 0.93^b^	0.00889 ± 0.0004^b^	78.07 ± 3.58^a^
*Glutamicibacter nicotianae* (NH27)	0.100	0.09413 ± 0.0022^c^	5.87 ± 2.16^a^	0.00202 ± 0.0008^a^	389.94 ± 187.49^b^
Consortium (NH2 + NH27)	0.100	0.0732 ± 0.0009^a^	26.84 ± 0.94^c^	0.01042 ± 0.0004^c^	66.6 ± 2.77^a^

### SEM analysis of PVC-MPs

**Fig. 4:**
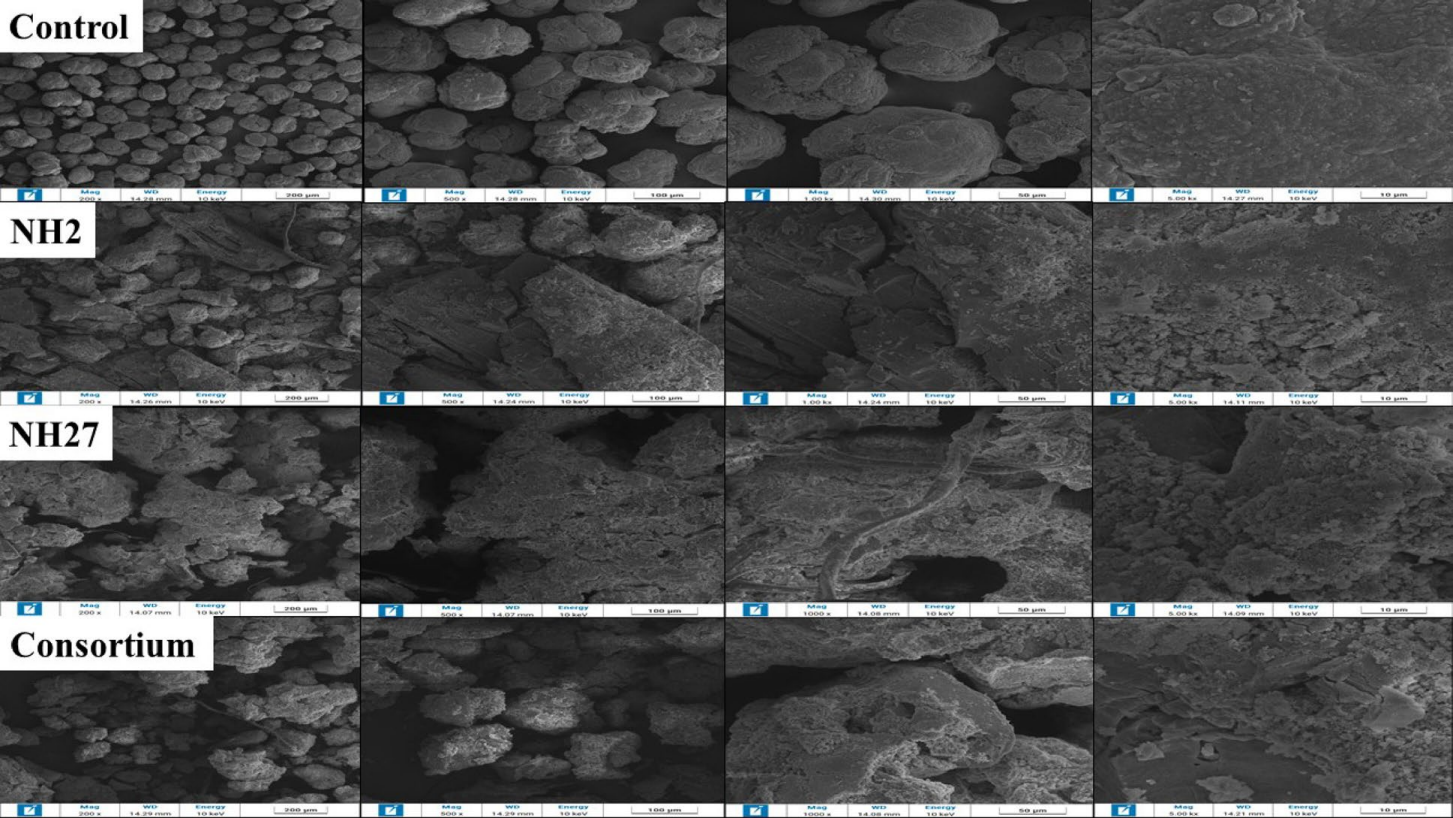
Scanning electron microscopy (SEM) images showing the surface morphology of MPs after 30 days of treatment. Images are presented at different magnifications, with the untreated control shown first, followed by samples treated with *Stutzerimonas* sp. NH2 and *Glutamicibacter nicotinae* NH27 and the consortium

Figure 4 illustrates SEM images of the PVC-MPs surface before and after biodegradation treatment. SEM analysis demonstrated microbial degradation activity on the MPs samples. The samples’ surfaces exhibited a loss of smoothness following the degradation process, with observable physical alterations including surface disruption, visible MPs, cracks and pores, erosion, and grooves, attributed to bacterial colonization. Conversely, the SEM micrograph of the control exhibited a smooth surface under the same conditions. The highest incidence of degradation signs was noted on the consortium sample.

### Fourier transform infrared (FTIR) analysis

**Fig. 5:**
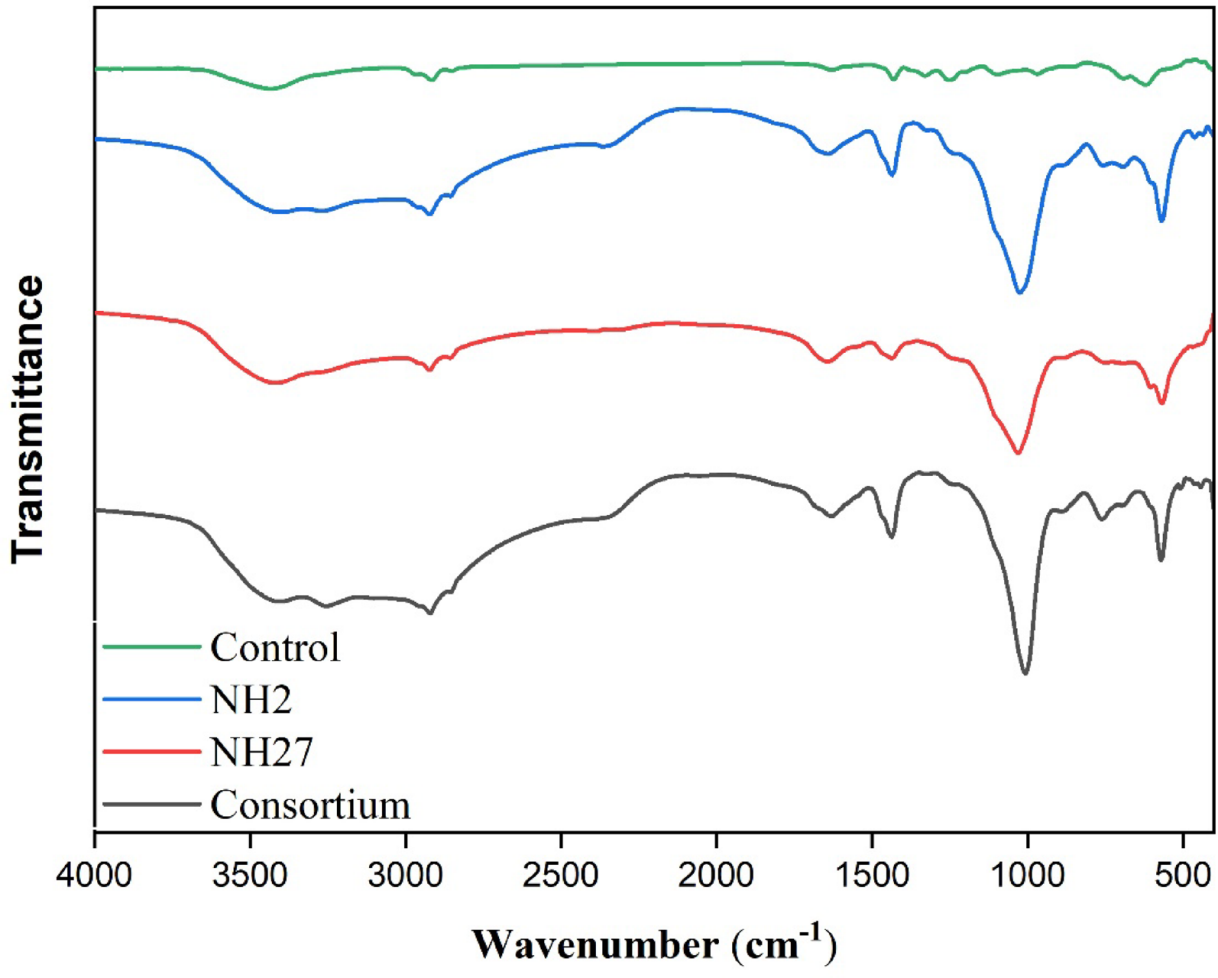
Fourier-transform infrared (FTIR) spectra of MPs after 30 days, including the untreated control, samples treated with *Stutzerimonas* sp. NH2 and *Glutamicibacter nicotinae* NH27 and the consortium

The FTIR spectrum exhibits the changes in the surface structure of the samples before and after treatment over the frequency range of 4000–500 cm⁻¹, as shown in Fig. 5. The leading characteristic absorption bands observed in the control group include a broad band at ~ 3420 cm⁻¹ corresponding to O–H stretching vibrations (hydroxyl groups) attributed to moisture absorption; a band at ~ 1720 cm⁻¹ assigned to C = O stretching of carbonyl groups formed during oxidation; a band at ~ 1630 cm⁻¹ associated with H–O–H bending of adsorbed water or C = C stretching in unsaturated segments; a band at ~ 1332 cm⁻¹ related to C–H deformation of CHCl groups; a band at ~ 1256 cm⁻¹ attributed to C–H wagging of CH₂–Cl groups; a band at ~ 1096 cm⁻¹ assigned to C–C stretching in the PVC backbone; a peak at ~ 959 cm⁻¹ corresponding to trans C–H wagging; a band at ~ 834 cm⁻¹ assigned to C–Cl stretching; and a band at ~ 616 cm⁻¹ corresponding to the strong C–H stretching mode. Compared with the control, the NH2- and consortium-treated samples exhibited marked reductions in the intensities of the C–H stretching (~ 2920 cm⁻¹) and C = O stretching (~ 1720 cm⁻¹) bands, indicating degradation of aliphatic and carbonyl structures, with the consortium showing the most pronounced changes. The NH27-treated sample showed moderate decreases in peak intensities while retaining most of the control’s original absorption features. In addition, a weak new band near 2360 cm⁻¹ appeared in the consortium-treated sample, which may correspond to C = O stretching or to CO₂ adsorption during testing. These spectral variations suggest that the treatments, particularly the consortium sample, induced structural modifications in the PVC through bond cleavage and the formation of new functional groups.

### Thermogravimetric analysis of PVC-MPs

**Fig. 6:**
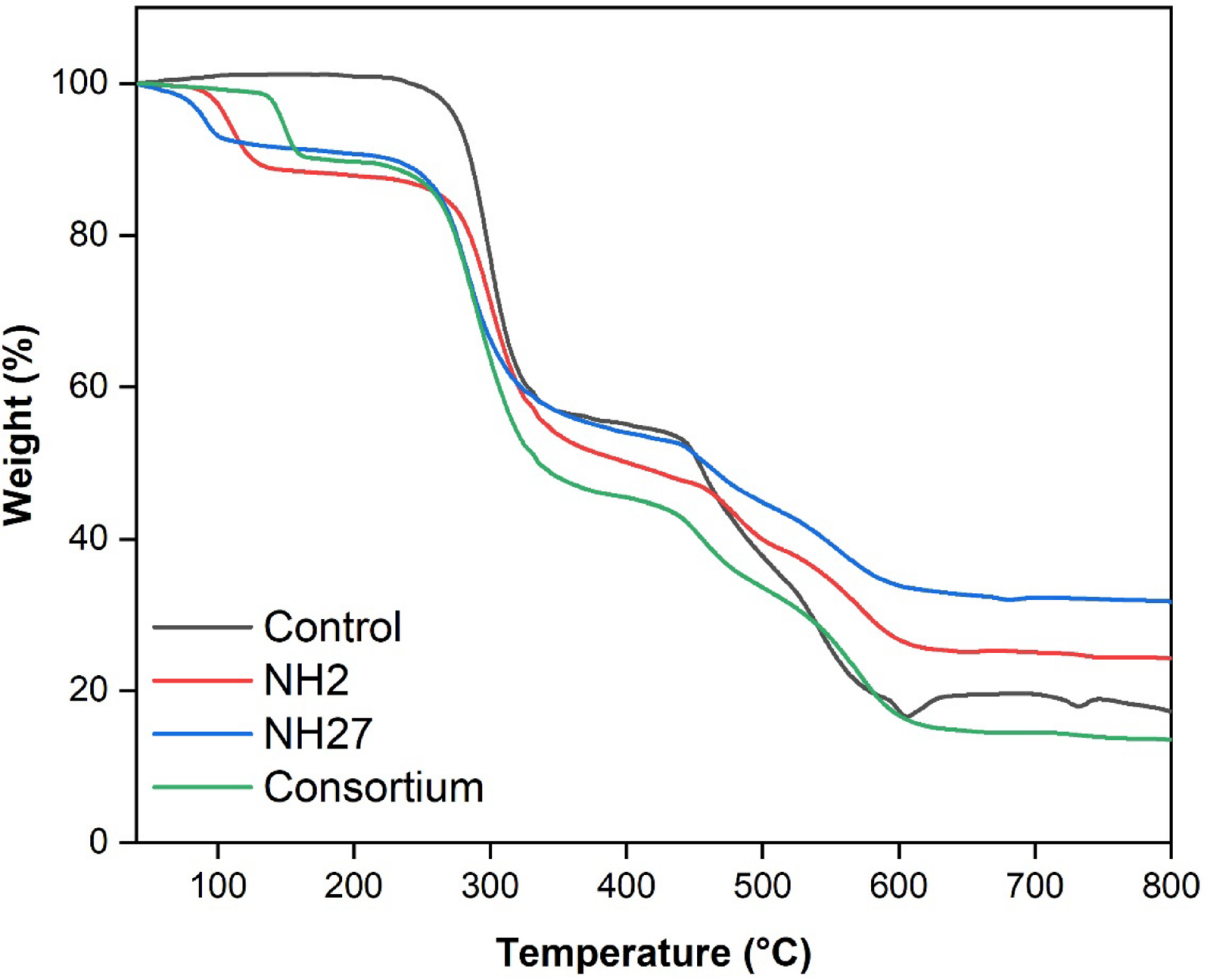
Thermogravimetric analysis (TGA) of MPs after 30 days, including the untreated control, samples treated with *Stutzerimonas* sp. NH2 and *Glutamicibacter nicotinae* NH27, and the consortium

Thermogravimetric analysis revealed that after 30 days, all samples exhibited multi-step degradation within the temperature range of 100–800 °C, as shown in Fig. 6. The results indicated that for the control group, the temperature at which 5% weight loss (T₅) occurred was approximately 250 °C. In contrast, sample NH2 exhibited a slightly higher T₅ of ~ 270 °C, indicating marginal improvement in thermal stability, whereas the consortium displayed a lower T₅ (~ 200 °C), reflecting reduced stability. Sample NH27 showed an elevated onset of degradation, with T₅ around 330 °C, indicating enhanced thermal stability compared to the control. At 800 °C, the residual mass was approximately 15% for the control, 20% for NH2, 30% for NH27, and 15% for the consortium. These results indicate that the applied treatments notably altered the onset and rate of thermal degradation, with the consortium decreasing the thermal stability relative to the control.

### GC–MS analysis

GC–MS analysis revealed clear differences between control and consortium-treated PVC-MPs samples, as summarized in Table 2. The plasticizer bis(2-ethylhexyl) phthalate (DEHP) was detected in the control sample at a retention time (RT) of 19.693 min, while in the treated sample it appeared at an earlier RT of 17.036 min. Similarly, 1,2-benzenedicarboxylic acid esters were observed in the control within an RT range of 16–18 min, whereas in the treated sample, these compounds were detected at 10.526 min. Phenol, 2,4-bis(1,1-dimethylethyl)-, phosphite was detected at RT 22.998 min in the control and at 21.214 min in the treated sample. Benzoic acid was detected only after treatment at RT 6.433 min. Guaiacol was present in both samples, eluting at RT 6.22 min in the control and 6.206 min in the treated sample, with an increased relative abundance following bacterial treatment. Phenolic derivatives, specifically 3,5-bis(1,1-dimethylethyl)phenol, were detected only in the treated sample at RT 8.907 min. Glycerol was also observed in the treated sample, eluting at RT 7.129 min. Fatty acids were detected in both samples, with palmitic acid eluting at RT 15.347 min in the control and 13.521 min in the treated sample, and stearic acid appearing at RT 17.042 min and 15.325 min in the control and treated samples, respectively. The long-chain amid 13-docosenamide was present in both samples but shifted from RT 21.177 min in the control to 19.62 min in the treated sample, with reduced abundance.

**Table 2:** List of identified chemical compounds in GC–MS analysis, including the control (PVC-MPs + MSM) and bacterial-treated sample (PVC-MPs + MSM + consortium)

Compound	RT (min)		Observation
	Control	Treatment	
Bis(2-ethylhexyl) phthalate (DEHP) (Plasticizer)	19.693	17.036	Plasticizer
1,2-Benzenedicarboxylic acid esters (Plasticizer)	16–18	10.526	Plasticizer
Phenol, 2,4-bis(1,1-dimethylethyl)-, phosphite	22.998	21.214	Antioxidant & Stabilizer
Benzoic acid	-	6.433	Aromatic Compound
Guaiacol	6.22	6.206	Aromatic Compound
Phenolic derivatives (3,5-bis(1,1-dimethylethyl) phenol)	-	8.907	Phenolic Compound
Glycerol	-	7.129	Alcohol
Palmitic acid	15.347	13.521	Fatty Acid
Stearic acid	17.042	15.325	Fatty Acid
13-Docosenamide	21.177	19.62	Fatty Amide

## Discussion

PVC is one of the most persistent and polluting plastics in the environment. So far, no strategy has been established as a definitive solution to tackle this issue. In this context, biotransformation and biodegradation have gained attention as promising approaches, offering potential routes to reduce the environmental burden of this challenging pollutant [46].

This study demonstrated that a bacterial consortium composed of two strains degraded PVC-MPs. When compared to treatments with *Stutzerimonas* sp. NH2 or *Glutamicibacter nicotianae* NH27 individually, the combined consortium led to a significantly higher weight loss of PVC-MPs. Structural, morphological, and thermal analyses further confirmed that microbial treatments, especially those involving the consortium, caused notable alterations in PVC-MPs, consistent with previous reports on polymer degradation. Moreover, GC–MS analysis offered valuable insights into the potential degradation products formed during the treatment [29, 47, 48].

FTIR results indicated a substantial reduction in the intensities of the (C–H) and (C = O) stretching bands, indicating degradation of aliphatic chains and carbonyl groups, respectively. The variations, especially prevalent in the consortium-treated samples, indicate clear chemical modifications of polymer constituents. Notably, analogous findings were reported in a study using a bacterial consortium derived from *Tenebrio molitor* larvae, which revealed elevations in –OH and C = C groups, accompanied by decreases in chlorine concentration, indicating PVC bond breaking and functional group transformation [49]. Moreover, marine-derived bacterial consortia exhibited FTIR shifts in OH, C–H, and C–Cl regions, indicating surface-level breakdown of PVC-MPs films [50]. These shifts are direct proof that the microbial consortia elicited irreversible structural alterations via bond-breaking and the formation of distinct functional groups. SEM imaging revealed clear surface degradation, including cracks, grooves, and pores, particularly in samples treated with the consortium. These alterations confirm that the consortium’s degrading efficacy surpassed that of the individual bacterial strains, likely due to synergistic metabolic processes. This investigation substantiates the degradation of microplastics by bacteria and validates the microbial capacity to decompose these substances. These findings align with the extensive morphological disturbances and robust biofilm formation observed in high-efficiency consortia studies, which demonstrate clear surface degradation and biofilm formation on plastic polymers [19, 49]. Such localized disturbances indicate not only active bacterial colonization but also the coordinated mechanical and enzymatic cleavage of the polymer chains, leading to the eventual disintegration of the PVC matrix [51]. The TGA profile showed variations in thermal behavior, with consortium treatment reducing thermal stability, suggesting significant polymer degradation. The consortium’s changes in stability further suggest chemical restructuring through bacterial activity. Similar results were observed with Streptomyces treatments, where bacterial exposure altered the stages of PVC degradation, as identified by TGA [46].

GC–MS analysis revealed notable changes in the chemical profile of PVC-MPs following exposure to the bacterial consortium. The presence of plasticizers alongside antioxidants and stabilizers in the control sample is common, as described earlier in similar MPs degradation studies [36]. Such plasticizers are added to enhance flexibility, and stabilizers or antioxidants are added to improve thermal and oxidative stability [52].

In the PVC-MPs sample treated with the bacterial consortium, small aromatic compounds, including benzoic acid, guaiacol, and other phenolic derivatives, were detected at high abundance. These compounds may represent secondary metabolites generated by bacterial metabolism, either constitutively or in response to available carbon sources. Their presence suggests partial transformation of the PVC polymer matrix or its associated additives, leading to the formation of oxidized and aromatic moieties, consistent with observations reported for other aliphatic synthetic plastics [46]. Fatty acids such as palmitic and stearic acids, found in both control and treated samples, may originate from additives in the PVC matrix or from microbial metabolism. Additionally, changes in the abundance of other compounds, including 13-docosenamide and phosphite derivatives, further indicate active microbial transformation [46, 53].

Overall, these spectroscopic, morphological, and thermal results confirm that microorganism treatments, particularly the consortium, clearly degrade the integrity of PVC-MPs. They promote bond cleavage, functional group transformation, and structural degradation, mirroring extensive degradation measures observed in several studies [43, 54, 55].

Although several bacterial species have been reported to degrade PVC Polymers or alter their chemical structure, no studies have documented the biodegradation of PVC-MPs by *Stutzerimonas* sp. or *Glutamicibacter* sp. based on current literature. Therefore, to the best of our knowledge, this study represents the first report of PVC-MPs biodegradation using these bacterial taxa.

Further investigations are recommended to gain a deeper understanding of the pathways and enzymes involved in the biodegradation of PVC-MPs by the reported isolates. Such knowledge could guide efforts to enhance enzyme production and to discover new microbial species with the potential to degrade PVC. In addition, whole-genome sequencing could help determine whether these species share genes associated with PVC-MPs degradation. Altogether, these efforts pave the way for sustainable and effective solutions to mitigate plastic pollution.

## Conclusion

The present study represents the first report on the biodegradation of PVC-MPs by the strain *Stutzerimonas* sp. NH2 and *Glutamicibacter nicotianae* NH27. Although both individual strains induced structural and chemical modifications in PVC-MPs, the bacterial consortium exhibited significantly higher degradation efficiency, reflecting potential cooperative microbial interactions. These findings establish the potential of the isolated strains and their combination for the biodegradation of PVC-MPs and provide a foundation for future mixed-culture strategies, offering a sustainable approach to mitigating persistent plastic pollution.

## Data Availability

No datasets were generated or analysed during the current study.
